# Numerical Study on the Influence of Model Uncertainties on the Transport of Underwater Spilled Oil

**DOI:** 10.3390/ijerph19159274

**Published:** 2022-07-29

**Authors:** Daosheng Wang, Zhixuan Luo, Lin Mu

**Affiliations:** 1 Hubei Key Laboratory of Marine Geological Resources, China University of Geosciences, Wuhan 430074, China; wangds@cug.edu.cn (D.W.); 20141003081@cug.edu.cn (Z.L.); 2 College of Life Sciences and Oceanography, Shenzhen University, Shenzhen 518060, China; 3 Southern Marine Science and Engineering Guangdong Laboratory (Guangzhou), Guangzhou 511458, China; 4 Shenzhen Research Institute, China University of Geosciences, Shenzhen 518057, China

**Keywords:** underwater spilled oil, numerical simulation, model uncertainties

## Abstract

Oil pollution influences marine biology, ecology, and regional sustainable development capacity, but model uncertainties limit the ability of the numerical model to accurately predict the transport and fate of the underwater oil spill. Based on a three-dimensional underwater oil spill model validated by satellite images of the oil slick at the sea surface, the Penglai 19-3 oil spill accident in the Bohai Sea was simulated; in addition, several sensitivity experiments were set up to investigate the influence of model uncertainties in the background wind, current, start time of the oil spill, and spill site on the transport of underwater spilled oil in the Penglai 19-3 oil spill accident. The experimental results indicate that the uncertainty in the background wind has a certain impact on the simulated centroid position at the sea surface, and little effect on the simulated underwater results, while the uncertainty in the background current has a significant influence on the transport of the underwater spilled oil both at the sea surface and underwater. An uncertainty of 24 h in the start time of the oil spill can cause more than 1 time larger than the benchmark case displacement of the oil spill centroid point and sweeping area at the sea surface, as the periodic tidal current is the main constituent of the ocean current in the Bohai Sea. The uncertainty in the spill site has a large influence on the final position of the oil spill centroid point, but the oil spill trajectories do not intersect with each other within 48 h, which makes it possible to identify the oil spill platform from the actual observations. The influence of uncertainties in the important model inputs and key model parameters on the transport of underwater spilled oil in the Penglai 19-3 oil spill accident is evaluated for the first time, which is of substantial significance for improving the prediction accuracy of the transport and fate of underwater oil spills.

## 1. Introduction

Oil spill accidents have occurred frequently, seriously affecting the marine environment, beaches, and human activities [1,2,3]. In April 2010, the Deepwater Horizon drilling rig in the Gulf of Mexico exploded and the oil spilled continually. This incident was the most serious oil spill accident in the United States and caused substantial environmental and economic losses. In June 2011, oil spills occurred near the Penglai 19-3 (PL19-3) oilfield in the Bohai Sea [4], which seriously affected aquaculture fisheries, natural fishery resources, and marine ecology. It is necessary to obtain information about the oil spill accidents and predict the areas contaminated by oil spills at the first time. However, it is difficult to observe the spilled oil in a timely manner, especially the underwater spilled oil. Therefore, an accurate prediction of the transport of the underwater spilled oil using numerical models is significant for oil spill risk-assessment and emergency response during the oil spill accidents [5].

As indicated by Yang et al. [6], three generations of oil spill models, including the empirical transport model, the two-dimensional fate model based on hydrodynamic model, and the three-dimensional transport and fate model, have been developed and used to investigate the fate and impact of oil spills. Early research has mainly focused on oil spills at the sea surface or near the sea surface [7,8]. Subsequently, underwater oil spill models have been developed to investigate the behavior of underwater oil spills [9,10,11,12,13]. The Eulerian method, Lagrangian method, Eulerian–Lagrangian method, and lattice Boltzmann method have been applied to simulate the transport of the spilled oil [6]. However, numerical models cannot provide perfect simulation and prediction results [14], due to numerical errors and model uncertainties, such as errors in the wind, current, and oil source [15].

An analysis of model uncertainties is required for reliable numerical simulation and prediction [16]. Elliott and Jones [17] indicated that the wind was a much more important requirement for simulating an oil slick than the current, buoyancy, and weathering processes. Jorda et al. [14] found that errors in the wind and current had an important impact on predicting the final oil position using the typical twin experiments. Yapa et al. [18] investigated the behavior of oil and gas spills in deep water and found that the oil droplet sizes were the most important factor impacting the amount of spilled oil on the sea surface or staying submerged. Li et al. [19] studied the impact of the vertical diffusion scheme on oil dispersion and transportation, and found that the scheme by solving the Langevin equation was the best one. Wang et al. [16] used the polynomial chaos expansions to analyze the uncertainties in six input parameters in an oil-gas plume model, and found the sensitive and insensitive parameters. Gonçalves et al. [5] analyzed the effect of the uncertainties in ocean current and oil droplet size data on the simulated oil concentration in the oil-fate model using a nonintrusive polynomial chaos method. Iskandarani et al. [20] analyzed the model uncertainties of an integral plume model by calculating the influence of five uncertain parameters, including entrainment coefficient, entrainment ratio, gas-to-oil rate, 95th percentile of the droplet size, and droplet distribution spreading ratio, on the simulated trap height and peel height. Hou et al. [21] developed the forecast uncertainty probability map and the HyosPy forecast reliability assessment to quantify the oil spill forecast uncertainty and assess the reliability of the oil spill forecast, respectively. They used these methods to study the accuracy and confidence intervals of the oil spill forecasts with the predicted wind and current. Li et al. [15] investigated the influence of the error in ocean current on the three-dimensional transport of the underwater spilled oil and gave a possible interpretation. Feng et al. [22] proposed a data-driven uncertainty model and a multi-model integration method to operationally quantify the effect of hydrodynamic model structural errors introduced by coarse grid solution on the prediction of oil transport paths. Yang et al. [6] provided a review of the decision support tools for oil spill response, and pointed out that it was necessary to deeply investigate the modeling oil spill because of the uncertainties in the background environment field.

As we know, when the underwater spilled oil reaches the sea surface and is found by observation devices or by people, the oil spill accident has already started for some time. The start time and spill site of the underwater oil spill are often not known when predicting the transport of the oil spill. In addition, the background winds and currents have different characteristics in the different areas. The uncertainties in the wind, current, start time of the oil spill, and oil spill site will influence the simulated transport of the underwater oil spill, especially for serious accidents. In this study, based on the underwater oil spill model developed by Chen et al. [9], the PL19-3 underwater oil spill accident in the Bohai Sea was simulated. A series of sensitivity experiments were carried out to investigate the influence of the uncertainties in the wind, current, start time of the oil spill, and oil spill site on the simulation results. In group 1 (group 2) of the sensitivity experiments, the artificial random perturbations were added in the wind (wind and current) to study the influence of the uncertainty in the wind (current). In group 3 and group 4 of the sensitivity experiments, the start time and spill site of the PL19-3 oil spill accident were changed to investigate their impact. Through the results of these experiments, the influence of model uncertainties on the transport of underwater spilled oil in the PL19-3 oil spill accident in the Bohai Sea was studied for the first time, with the aim of providing significant information on the model parameters that need attention for the accurate prediction of serious oil spill accidents in future research.

## 2. Materials and Methods

### 2.1. Study Site

**Figure 1:**
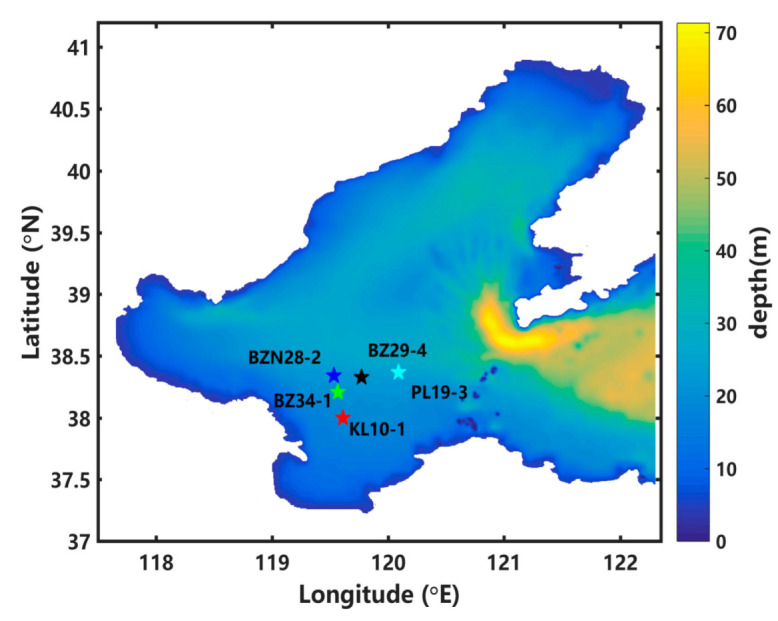
Depth map of the Bohai Sea and distribution of the oil platforms.

The Bohai Sea is a semi-closed shelf sea with a total area of 77,000 km^2^, situated in northeast China from 37° N to 41° N and from 117° E to 121° E [23,24], as shown in Figure 1. The wind in the Bohai Sea is dominated by the Asian monsoon, with a maximum velocity of >11 m/s in the winter [24]. Ocean tide is the major dynamic processes in the Bohai Sea [25].

As it has the second largest oil field and many oil resources, more than 50 offshore oil platforms have been established in the Bohai Sea [26]. Several oil platforms, including the PL19-3 oil platform, Bozhong 29-4 (BZ29-4) oil platform, Bozhong 34-1 (BZ34-1) oil platform, Bozhongnan 28-2 (BZN28-2) oil platform and Kenli 10-1 (KL10-1) oil platform, are in the central Bohai Sea (Figure 1). Various oil spill accidents have occurred in the Bohai Sea, including the PL19-3 oil spill accident, the “7–16” explosion of an oil pipeline in Dalian, and a pipeline blast in Qingdao in 2013. The PL19-3 oil spill accident occurred at the platforms of the PL19-3 oil field (120.08° E, 38.37° N) on 4 and 17 June 2011, and was the worst oil spill accident in China, which polluted about 840 km^2^ of sea water [27] and caused catastrophic economic loss and environmental pollution. Five years after this accident, the marine environment within a 2.2 km radius of the spill site remained a concern [26]. So, the PL19-3 oil spill accident was simulated in this study and used as a test case to investigate the influence of model uncertainties in underwater oil spill models on the transport of underwater spilled oil.

### 2.2. Underwater Oil Spill Model

According to Zheng et al. [13], the dynamic processes of an underwater oil spill include the turbulent jet stage, the buoyant plume stage, and the advection-diffusion stage. The first two stages can be simulated by the plume dynamics model, and the third stage can be simulated by the advection-diffusion model, as indicated in Chen et al. [9]. The criterion of the transition from the first two stages to the third is the neutral buoyancy level [28], i.e., when the plume density is equal to the density of the ambient seawater, the spilled oil will enter the advection-diffusion stage. Besides, the oil weathering processes also need to be considered. On the whole, the underwater oil spill model contains three sub-models, including the plume dynamic model, the advection-diffusion model, and the oil weathering model.

#### 2.2.1. Plume Dynamic Model

The plume dynamic model adopts the Lagrange integral technique, in which the oil spill duration is divided into several parts with the same time interval. Therefore, the spilled oil at the turbulent jet stage and the buoyant plume stage is assumed to be a bent cone, and is divided into a series of non-interfering elements according to the time interval [9]. Each element is assumed to be a cylinder with its properties, such as radius, local velocity, density, temperature, and oil concentration. Those properties will be changed as the element moves from the wellhead to the terminal level of plume dynamics.

During the turbulent jet and buoyant plume stages, the elements will maintain the conservation of mass, momentum, heat, salinity, and oil concentration. In addition, oil density variation due to temperature and the entrainment processes are also considered. The details of the plume dynamic model can be found in Chen et al. [9].

#### 2.2.2. Advection-Diffusion Model

The advection-diffusion model adopts the Lagrangian particle-tracking method, in which the spilled oil is taken as a collection of oil particles. The distribution of oil particle size is estimated according to Delvigne and Hulsen [29], Wang et al. [30], and Cao et al. [31]. The Lagrangian particle-tracking method is effective in simulating the advection-diffusion process as shown in Al-Rabeh et al. [32], Yapa et al. [28], and Wang et al. [30]. For each oil particle, the three-dimensional advection-diffusion model is described as follows:

(1) $$\frac{d\vec{S}}{dt}=\left\{ \begin{array}{l} {\vec{U}}_{current}+{\vec{U}}_{diffusion}+{\vec{U}}_{wind},\;\;\mathrm{at}\;\mathrm{sea}\;\mathrm{surface}\\ {\vec{U}}_{current}+{\vec{U}}_{diffusion}+w_{b}\vec{k},\;\mathrm{underwater} \end{array}\right.$$

where $\vec{S}=\left(x,y,z\right)$ is the displacement of oil particle; x, y and z are Cartesian coordinates; ${\vec{U}}_{current}$ is the velocity of the oil particle due to the effect of the ocean current, which is equal to the three-dimensional velocity of the ocean current; ${\vec{U}}_{diffusion}$ is the diffusion velocity of the oil particle due to the turbulent diffusion process; ${\vec{U}}_{wind}$ is the velocity of the oil particle due to the effect of wind; *w_b_* is the buoyancy velocity of oil particle and is calculated with the oil droplet size using the methods in Yapa et al. [28]; $\vec{k}$ is the unit vector in the vertical direction.

The diffusion velocity ${\vec{U}}_{diffusion}$ of the oil particle is calculated by the random walk method, as follows:

(2) $${\vec{U}}_{diffusion}=\left(u_{diffusion},v_{diffusion},w_{diffusion}\right)=\sqrt{\frac{6}{\Delta t}}\left(R_{x}\sqrt{K_{x}},R_{y}\sqrt{K_{y}},R_{z}\sqrt{K_{z}}\right)$$

where *u_diffusion_*, *v_diffucion_* and *w_diffusion_* are the components of the diffusion velocity in the *x*, *y* and *z* directions, respectively; *K_x_*, *K_y_* and *K_z_* are the components of the dispersion coefficient in the *x*, *y* and *z* directions, respectively, which can be calculated as described by Cao et al. [31]; *R_x_*, *R_y_* and *R_z_* are independent random numbers ranging from −1 to 1 with uniform distribution; Δt is the time step of the advection-diffusion model.

${\vec{U}}_{wind}$ is calculated as follows:

(3) $${\vec{U}}_{wind}=\left(u_{wind},v_{wind}\right)=\left(\alpha u_{10}\cos\beta +\alpha v_{10}\sin\beta ,-\alpha u_{10}\sin\beta +\alpha v_{10}\cos\beta \right)$$

where *u_wind_* and *v_wind_* are the components of the oil drift velocity at the sea surface due to wind in the *x* and *y* directions, respectively; *u*_10_ and *v*_10_ are the components of the wind velocity at 10 m above sea surface in the *x* and *y* directions; *α* is the wind drift factor; *β* is the deviation angle, which is $40-8\sqrt[{4}]{u_{10}^{2}+v_{10}^{2}}$ when the wind speed $\sqrt{u_{10}^{2}+v_{10}^{2}}$ is not larger than 25 m/s and is 0 otherwise.

#### 2.2.3. Oil Weathering Model

Besides the dynamic processes of the underwater oil spill, the spilled oil is also affected by the weathering processes. The spilled oil will be dissolved into the ambient environment and the mass is lost. In addition, the spilled oil at the sea surface will be evaporated into the air and the mass will be rapidly reduced. Besides, emulsification can also affect the mass and constituent of the spilled oil. The dissolution, evaporation, emulsification, and density variation of the spilled oil are simulated by the oil weathering model. The details of the oil weathering model can be found in Chen et al. [9] and Li et al. [15], and will not be repeated here.

### 2.3. Model Validation

The background wind and current are the important inputs of the underwater oil spill model, which are provided by Li et al. [15]. The wind was obtained from NCEP Climate Forecast System Version 2 (CFSv2), with a horizontal resolution of 0.5° × 0.5° and temporal resolution of 6 h. FVCOM was used to simulate the background hydrodynamic field in the Bohai Sea with the following model settings. The whole area ranged from 117.5° E to 122.3° E and from 37° N to 41° N (Figure 1). The horizontal resolution was 1/24° and the water column was equally divided into 10 sigma layers in the vertical direction. Water depth was obtained from the General Bathymetric Chart of the Oceans, with a horizontal resolution of 0.5′ × 0.5′. The longwave radiation and latent heat flux were obtained from NCEP CFSv2. In addition, the temperature decreased from the sea surface to the bottom, with 25 °C at the sea surface and 15 °C at 75 m. The salinity was specified as 30 psu. The open boundary conditions were set to eight tidal constituents ($M_{2}$, $S_{2}$, $N_{2}$, $K_{2}$, $K_{1}$, $O_{1}$, $P_{1}$ and $Q_{1}$) and the harmonic constants of these constituents were obtained from a tidal model with the adjoint data assimilation [33,34]. The simulated background current is in satisfactory agreement with the tidal level and the tidal current observations, as shown in Li et al. [15].

**Figure 2:**
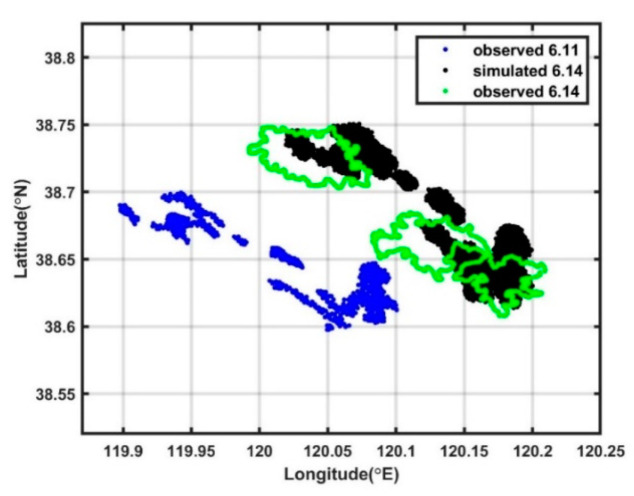
Distribution of oil slick at the sea surface observed on 11 June (blue dots) and 14 June (green dots), and the simulated result for 14 June (black dots).

As shown in Xu et al. [4], the PL19-3 oil spill accident in the Bohai Sea was captured in satellite images. The satellite images at 02:14:57 on 11 June 2011 and at 02:05:02 on 14 June 2011 were used to validate the underwater oil spill model. The plume dynamic model was turned off, and the first satellite image was used as the initial distribution of the spilled oil at the sea surface for the underwater oil spill model. Thus, the transport of the oil slick was simulated using the advection-diffusion model and the oil weathering model from 02:00:00 on 11 June to 02:00:00 on 14 June with a 30 s time step. As shown in Figure 2, the simulated distribution of the oil slick at the sea surface at 02:00:00 on 14 June 2011 is close to that captured by the satellite image at 02:05:02 on 14 June 2011, indicating the reliability of the underwater oil spill model.

### 2.4. Experimental Design

In benchmark experiment Exp1, the PL19-3 oil spill accident in the Bohai Sea was simulated using the underwater oil spill model. The PL19-3 oil spill site was located at the bottom of PL19-3 platform (120.08° E, 38.37° N) with a depth of 24 m. The spill was assumed to start at 12:00 on 17 June 2011, and last for 1 h. The model duration was set to 48 h. The nozzle diameter was assumed to be 0.46 m and the initial eruption direction of the oil spill was vertically upward with spill velocity of 0.4 m/s. The oil density was set to 950 kg/m^3^ and the spilled oil was divided into 100,000 oil particles. For reducing the influence of the turbulent diffusion process, 100 scenarios, with different seeds when generating the random numbers in the random walk method, were conducted in Exp1.

Model uncertainties, including the uncertainty in background wind, background current, start time of the oil spill, and spill site, are important factors affecting the simulated results of underwater oil spills. Four groups of sensitivity experiments were designed to study the influence of the uncertainties on the simulated results. As simulated by the numerical model, the background wind and current are usually inaccurate, contributing to the uncertainties of the underwater oil spill model. In the sensitivity experiments numbered with the prefix ‘SE1’, the influence of the uncertainty in background wind on the simulation results was discussed by adding artificial random perturbations in the background wind. In sensitivity experiments SE1_1, SE1_2, and SE1_3, the maximum random errors were 10%, 20%, and 30%, respectively. The artificial random perturbations were added into the background wind using Equation (4), as follows:

(4) $$uv=uv_{sim}\times \left[1+\left(-1+2r\right)\times e\right]$$

where *uv_sim_* is the wind speed (current speed) simulated by the numerical model as used in Exp1, *r* is the random number ranging from 0 to 1 with uniform distribution, *e* is the maximum random error, and *uv* is the wind speed (current speed) used in the corresponding sensitivity experiments.

In the sensitivity experiments numbered with the prefix ‘SE2′, the influence of the uncertainty in the background current on the simulation results was discussed by simultaneously adding artificial random perturbations in the background wind and current by using Equation (4). In sensitivity experiments SE2_1, SE2_2, and SE2_3, the maximum random errors were 10%, 20%, and 30%, respectively. It was noted that the random numbers were generated using different seeds when adding artificial random perturbations into the wind and current.

In sensitivity experiments numbered with ‘SE3′, the influence of the uncertainty in the start time of the oil spill on the simulation results was discussed. Compared with Exp1, the start time of the oil spill was delayed from 1 to 24 h; in addition, the model duration was 24 h.

In sensitivity experiments numbered with the prefix ‘SE4′, the influence of the spill site uncertainty on the simulation results was discussed. In sensitivity experiments SE4_1, SE4_2, SE4_3, and SE4_4, the site of the oil spill was changed from PL19-3 to BZ29-4, BZ34-1, BZN28-2, and KL10-1, respectively.

The detailed model settings of the sensitivity experiments are listed in Table 1, and the other model settings are the same as those in Exp1. Similar to that in Exp1, 100 scenarios, with different seeds when generating the random numbers in the random walk method, and adding the artificial random perturbations, were conducted in all the sensitivity experiments. The simulated centroid point, sweeping area, and sweeping volume were analyzed to show the transport of the spilled oil. In detail, the sweeping area is the area where the water has been contaminated, and the sweeping volume is the total volume polluted by the oil spill since the start. According to Li et al. [15], the influence of the uncertainties is evaluated by calculating the error expectation and result uncertainty, as follows:

(5) $$EE\left(t\right)=\frac{\sum_{i=1}^{100}\left|R_{i}^{t}-R_{0}^{t}\right|}{100}$$

(6) $$RU\left(t\right)=\sqrt{\frac{\sum_{i=1}^{100}{\left|R_{i}^{t}-{\bar{R}}^{t}\right|}^{2}}{100}}$$

where *EE* is the error expectation, which is the average difference at time *t* between the simulated results of 100 scenarios in one experiment and the scenarios-average simulated result in Exp1; $R_{i}^{t}$ is the result, including centroid point, sweeping area, and sweeping volume at the sea surface or underwater, in each scenario at time *t*; $R_{0}^{t}$ is the scenarios-average simulated result in Exp1 at time *t*; *RU* is the result uncertainty, which is the standard deviation of the 100 simulated results at time *t* in one experiment, and indicates the dispersion degree because of the model uncertainty; ${\bar{R}}^{t}$ is the scenarios-average value of $R_{i}^{t}$.

## 3. Results

### 3.1. Simulated Results of the PL19-3 Oil Spill Accident

**Figure 3:**
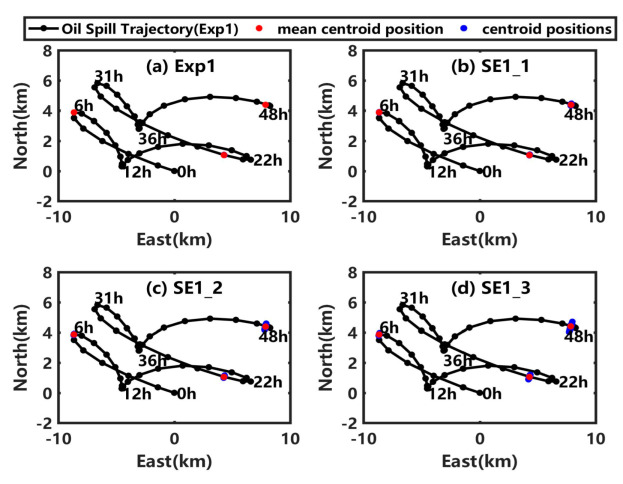
(**a**) Centroid positions of the simulated oil spill at the sea surface in 100 scenarios (blue dots) and the mean value (red dots) at 6 h, 24 h, and 48 h in Exp1. (**b**–**d**) are similar to (**a**), but for the sensitivity experiments SE1_1, SE1_2, and SE1_3. In each subplot, the black line with black dots is the averaged oil spill trajectory at the sea surface in Exp1.

The simulated centroid position and the oil spill trajectory in the PL19-3 oil spill accident obtained in Exp1 are shown in Figure 3a. The simulated centroid positions of the 100 scenarios in Exp1 are almost equal to the mean value, indicating the convergence of the results. As the transport time of oil/water plume is usually just several minutes [31], the spilled oil reaches the sea surface quickly and is transported by the wind and current. The tide is the main component of the ocean current in the Bohai Sea, so the oil spill trajectory at the sea surface changes direction nearly every 6 h, as shown in Figure 3a. The simulated results in Exp1 will be taken as the benchmark to calculate the influence of model uncertainties on the transport of underwater spilled oil in the sensitivity experiments.

### 3.2. Influence of Uncertainty in the Background Wind

**Figure 4:**
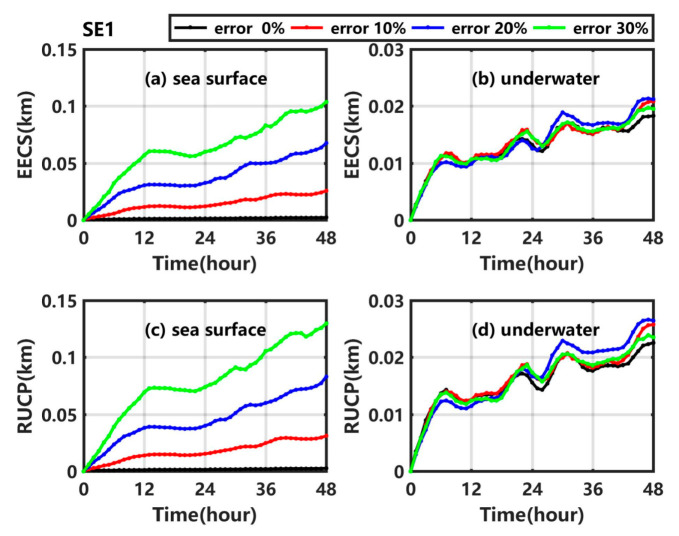
(**a**) EECS at the sea surface, (**b**) underwater EECS, (**c**) RUCP at the sea surface and (**d**) underwater RUCP in Exp1 (black line), SE1_1 (red line), SE1_2 (blue line), and SE1_3 (green line).

As shown in Figure 3, when the artificial random perturbations are added in SE1_1, SE1_2, and SE1_3, the oil spill trajectories in these sensitivity experiments are nearly the same as that in Exp1, but the dispersion degree of the centroid positions in 100 scenarios gets larger and larger as the maximum random error increases. The error expectation of centroid shift (EECS) and the result uncertainty of centroid position (RUCP) at the sea surface and underwater are calculated and shown in Figure 4. As shown in Figure 4a (Figure 4c), the EECS (RUCP) at the sea surface gradually increases with an increase of uncertainty in the wind from SE1_1 to SE1_3. The maximum values of the EECS (RUCP) at the sea surface in SE1_1, SE1_2, and SE1_3 are 0.03 km (0.03 km), 0.07 km (0.08 km), and 0.10 km (0.13 km), respectively, which is 10.17 times (10.51 times), 28.43 times (29.85 times), and 44.08 times (47.03 times) compared to that in Exp1, indicating that the uncertainty in wind would increase the deviation and uncertainty of the simulated centroid position at the sea surface. As shown in Figure 4b (Figure 4d), the underwater EECS (RUCP) also increases with time in SE1_1, SE1_2, and SE1_3, but the shift degrees (uncertainty degrees) of the underwater centroid position are almost the same in those experiments, because the wind could only drive the oil drift at the sea surface as shown in Equation (1).

**Figure 5:**
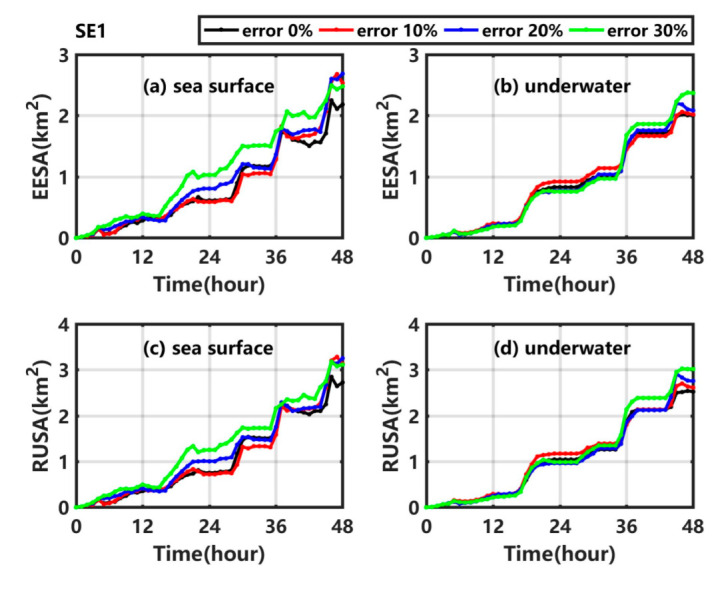
(**a**) EESA at the sea surface, (**b**) underwater EESA, (**c**) RUSA at the sea surface and (**d**) underwater RUSA in Exp1 (black line), SE1_1 (red line), SE1_2 (blue line), and SE1_3 (green line).

The error expectation of sweeping area (EESA) and the result uncertainty of sweeping area (RUSA) at the sea surface and underwater are calculated and shown in Figure 5. As shown in Figure 5a (Figure 5c), the EESA (RUSA) at the sea surface gradually increases with time and shows a step-like growth trend in SE1_1, SE1_2, and SE1_3, which may be related to the fact that the temporal resolution of wind is 6 h. As the uncertainty in the wind increases, the EESA and RUSA at the sea surface in SE1_3 are larger than those in the other experiments, but the results in SE1_1 and SE1_2 are not significantly different from those in Exp1. Below the sea surface, the underwater EESA (RUSA) values in SE1_1, SE1_2, and SE1_3 gradually increase over time and reach a maximum at 48 h, with values of 2.02 km^2^ (2.61 km^2^), 2.08 km^2^ (2.75 km^2^), and 2.38 km^2^ (3.01 km^2^), respectively. As the uncertainty in the background wind increases from SE1_1 to SE1_3, the average errors and uncertainty degrees of the underwater sweeping area show no significant difference (Figure 5b,d).

**Figure 6:**
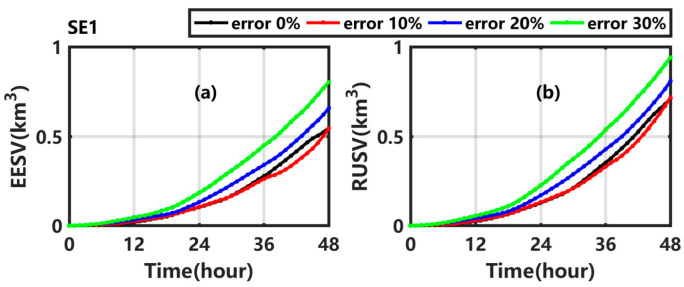
(**a**) EESV and (**b**) RUSV in Exp1 (black line), SE1_1 (red line), SE1_2 (blue line), and SE1_3 (green line).

The error expectation of sweeping volume (EESV) and the result uncertainty of sweeping volume (RUSV) are calculated and shown in Figure 6. The EESV (RUSV) gradually increases with time in SE1_1, SE1_2, and SE1_3; in addition, the maximum value at 48 h is 0.54 km^3^ (0.72 km^3^), 0.66 km^3^ (0.81 km^3^), and 0.80 km^3^ (0.94 km^3^). With the increase of the uncertainty in wind, the average error (uncertainty degree) of the sweeping volume increases by 0.00 times (0.01 times), 0.12 times (0.14 times), and 0.47 times (0.32 times) in SE1_1, SE1_2, and SE1_3, respectively, compared with the value in Exp1.

Overall, when the maximum random error in wind is 30%, the deviation of the simulated centroid position at the sea surface is about 0.10 km, and EECS at sea surface is 44.08 times that in Exp1; however, the other differences of underwater centroid position, sweeping area, and sweeping volume are insignificant, showing that the uncertainty in the background wind has a certain impact on the simulated centroid position at the sea surface, and little effect on the simulated underwater results.

### 3.3. Influence of Uncertainty in the Background Current

**Figure 7:**
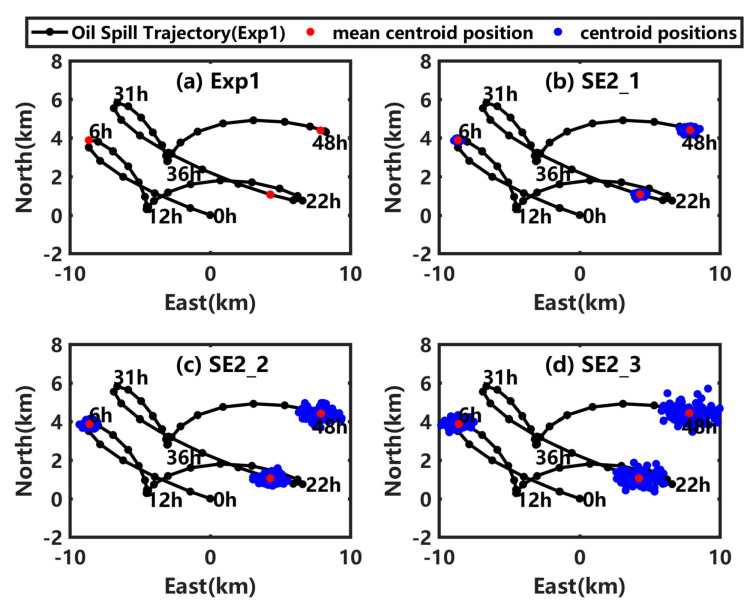
(**a**) Centroid positions of the simulated oil spill at the sea surface in 100 scenarios (blue dots) and the mean value (red dots) at 6 h, 24 h and 48 h in Exp1. (**b**–**d**) are similar to (**a**), but for the sensitivity experiments SE2_1, SE2_2, and SE2_3. In each subplot, the black line with black dots is the averaged oil spill trajectory at the sea surface in Exp1.

When the artificial random perturbations are simultaneously added into the background wind and current in SE2_1, SE2_2, and SE2_3, the simulated mean centroid positions in the 100 scenarios are near to those in Exp1, but the dispersion degree of those centroid positions increases, as shown in Figure 7. The distance between the simulated mean centroid position at 48 h in SE2_3 and that in Exp1 is 0.06 km, while the maximum distance for the 100 scenarios in SE2_3 is 2.63 km. In fact, the background current and wind are provided by the numerical model and the simulation error is unavoidable. As one of the scenarios in SE2_1, SE_2, and SE2_3 may be the used background current and wind in the practical application, the possible simulation error of the centroid position at the sea surface at 48 h is about 2.63 km, rather than 0.06 km. This is also the reason to analyze the error expectation of the centroid shift.

**Figure 8:**
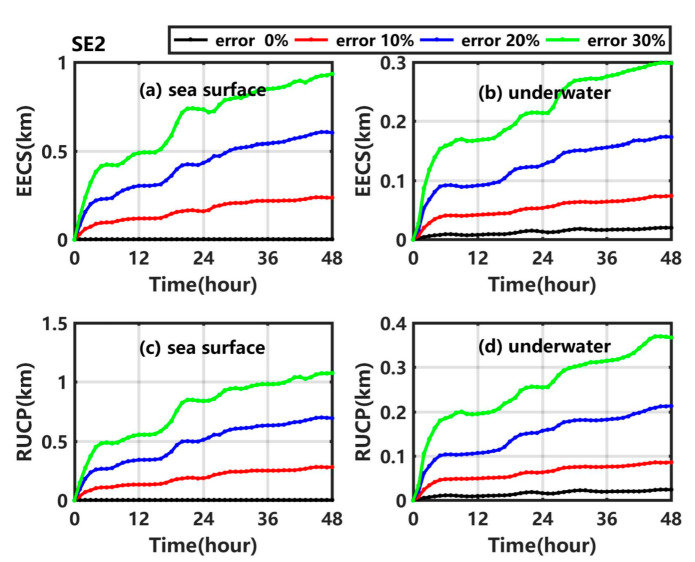
(**a**) EECS at the sea surface, (**b**) underwater EECS, (**c**) RUCP at the sea surface and (**d**) underwater RUCP in Exp1 (black line), SE2_1 (red line), SE2_2 (blue line), and SE2_3 (green line).

As shown in Figure 8a (Figure 8c), the EECS (RUCP) at the sea surface shows a gradual increasing trend in SE2_1, SE2_2, and SE2_3, and reaches maximum values of 0.24 km (0.28 km), 0.61 km (0.70 km), and 0.93 km (1.08 km), respectively, which are much larger than those in the corresponding sensitivity experiments numbered with the prefix ‘SE1′. With an increase of the uncertainty in the background wind and current from SE2_1 to SE2_3, the shift degree (uncertainty degree) of the underwater centroid position increases, as shown in Figure 8b (Figure 8d), which is much more obvious than when the artificial random perturbations are only added in wind. The results indicate that the uncertainty in the background current has a large impact on the shift degree and uncertainty degree of the centroid position, whether at the sea surface or underwater, which is significantly greater than that for the background wind.

**Figure 9:**
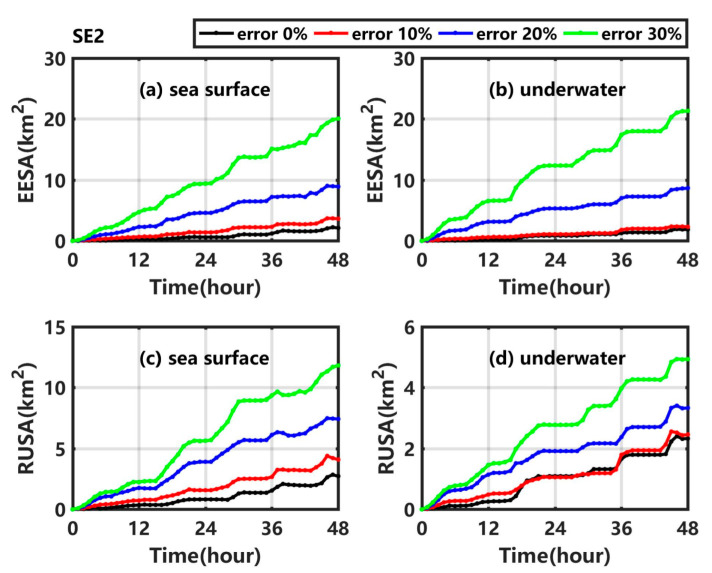
(**a**) EESA at the sea surface, (**b**) underwater EESA, (**c**) RUSA at the sea surface and (**d**) underwater RUSA in Exp1 (black line), SE2_1 (red line), SE2_2 (blue line), and SE2_3 (green line).

Figure 9a (Figure 9c) shows that the EESA (RUSA) at the sea surface gradually increases in SE2_1, SE2_2, and SE2_3. In addition, the larger the error, the larger the growth trend. The EESA (RUSA) at the sea surface at 48 h in SE2_1, SE2_2, and SE2_3 is 0.68 times (0.54 times), 3.09 times (1.63 times), and 8.09 times (3.1 times) higher than in Exp1. In SE2_1, SE2_2, and SE2_3, the underwater EESA (RUSA) also gradually increases with time, and its values at 48 h are much larger than that in Exp1. The results are significantly higher than those in the corresponding experiments numbered with the prefix ‘SE1′, especially when the maximum random error is 30%.

**Figure 10:**
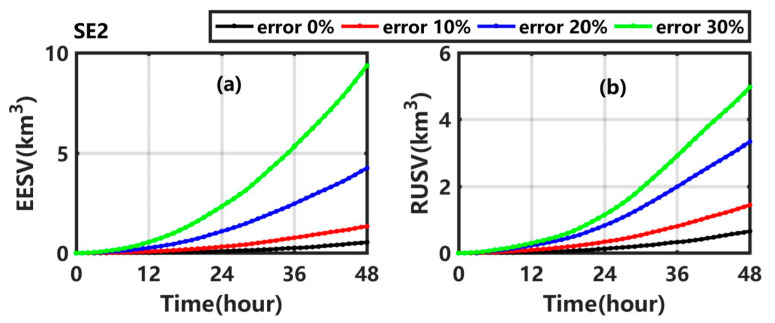
(**a**) EESV and (**b**) RUSV in Exp1 (black line), SE2_1 (red line), SE2_2 (blue line), and SE2_3 (green line).

Figure 10a (Figure 10b) shows that the EESV (RUSV) gradually increases in SE2_1, SE2_2, and SE2_3, and reaches 1.34 km^3^ (1.44 km^3^), 4.25 km^3^ (3.33 km^3^), and 9.36 km^3^ (4.97 km^3^), respectively. With the increase of the uncertainty in the background wind and current, the average error (uncertainty degree) of sweeping volume increases by 1.48 times (1.21 times), 6.87 times (4.10 times), and 16.33 times (6.61 times) relative to that in Exp1, which is significantly higher than the corresponding results in the experiments numbered with the prefix ‘SE1’.

In summary, when the uncertainties in the background current and wind are present simultaneously, the influence on the simulated centroid position, sweeping area, and sweeping volume is significantly greater than when the only the uncertainty is in the background wind at the same error level. The influence of the uncertainty in the background current on the transport of the underwater oil spill is greater than that of the uncertainty in the background wind, which is important for forecasting the transport and fate of the underwater spilled oil.

### 3.4. Influence of Uncertainty in the Start Time of the Oil Spill

**Figure 11:**
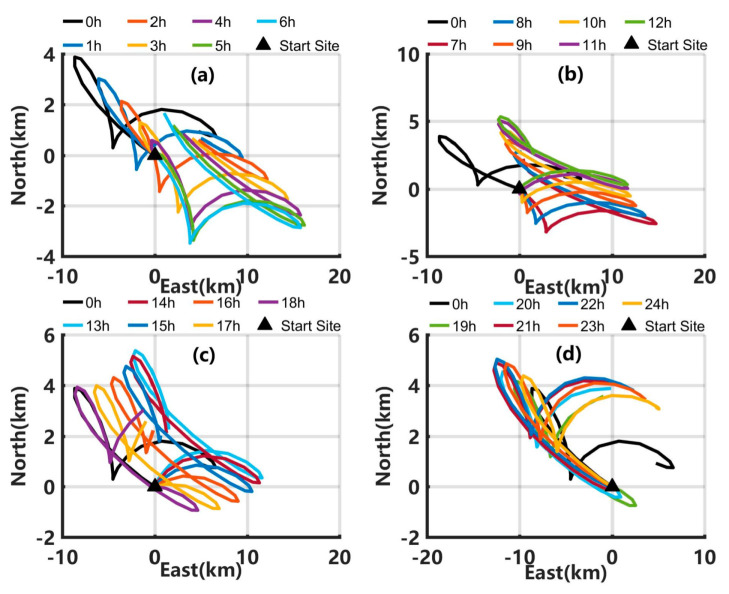
Centroid trajectory at the sea surface in Exp1 (black lines) and those in SE3 when the delay of start time is (**a**) 1 h to 6 h, (**b**) 7 h to 12 h, (**c**) 13 h to 18 h, and (**d**) 19 h to 24 h.

As the transport time of oil/water plume is usually just several minutes from well head to sea surface [31], several parts of the underwater spilled oil will reach the sea surface quickly. As shown in Figure 11, when the delay of start time is 1 to 4 h, the patterns of the centroid trajectory at the sea surface within 24 h are almost the same as in Exp1, but the centroid position gradually moves to the southeast. When the delay of start time is 5 to 10 h, the initial movement directions of the spilled oil at the sea surface are in the south by east direction, which is opposite to the direction in Exp1. When the delay is 11 to 17 h, the initial movement direction of the oil slick at the sea surface is northeast. When the delay is 22 to 24 h, the initial movement direction of the spilled oil at the sea surface, and the overall movement direction are almost the same as that in Exp1, but the reachable distance in the northwest direction is much longer.

**Figure 12:**
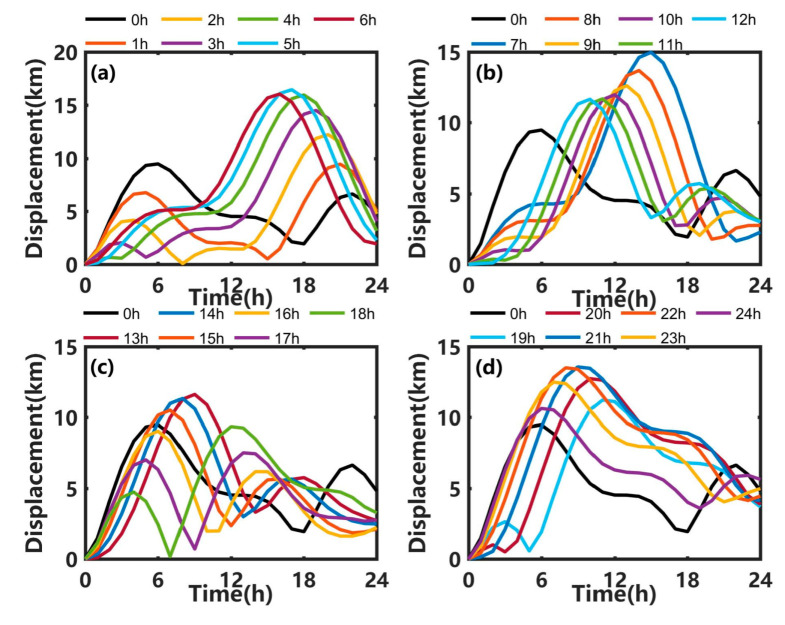
Centroid displacement at the sea surface in Exp1 (black lines) and SE3 when the delay of start time is (**a**) 1 h to 6 h, (**b**) 7 h to 12 h, (**c**) 13 h to 18 h, and (**d**) 19 h to 24 h.

As shown in Figure 12, the displacements of the oil centroid point at the sea surface in Exp1 and SE3 exhibit a typical double-peak structure. In Exp1, the maximum displacement is 9.48 km at 7 h. When the oil spill start time is delayed by 5 h, the maximum displacement is 16.44 km at 18 h, and the time to reach a displacement of 9.48 km is delayed by about 7 h compared with Exp1, meaning that there is enough time for emergency disposal of the oil slick at the sea surface when the oil spill start time is delayed by 5 h, but if the disposal is not timely, the scope of the oil spill will increase. Only when the oil spill start time is delayed by 16 to 18 h, the maximum displacements are lower than in Exp1.

**Figure 13:**
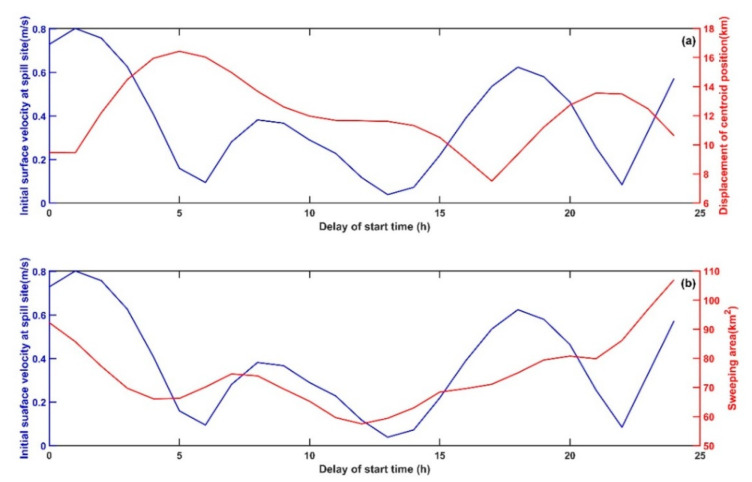
(**a**) The maximum displacement of the oil spill at the sea surface (red line) and the initial surface velocity at the location of oil spill site (blue line) in SE3. (**b**) The maximum sweeping area of the oil spill at the sea surface (red line) and the initial surface velocity at the location of oil spill site (blue line) in SE3.

As shown in Figure 13, with a change in the oil spill start time, the initial surface current velocity at the location of the oil spill site shows obvious periodic changes; in addition, the maximum displacement of the centroid position is negatively (−0.41) correlated with the initial surface current velocity, while the sweeping area is positively (0.50) correlated with the initial surface current velocity. When the start time of the oil spill is delayed, the maximum displacements of the centroid position in most cases are much larger than in Exp1. As shown in Figure 13a, the minimum displacement is 7.51 km when the delay is 17 h, while when the delay is 5 h the maximum value is 16.44 km, which is 2.19 times the minimum value. Figure 13b shows that with the passage of the oil spill start time, the sweeping area of the spilled oil at the sea surface generally shows a trend of first decreasing and then increasing, reaching a minimum of 59.64 ${\mathrm{km}}^{2}$ at a delay of 12 h and a maximum of 106.98 km^2^ at a delay of 24 h.

As discussed above, the uncertainty of the oil spill start time has a significant influence on the transport of the oil slick at the sea surface. An uncertainty of 24 h can cause 1.19 times larger than before displacement of the oil spill centroid point at the sea surface, and nearly 1-time larger than before sweeping area at the sea surface. Therefore, accurately knowing the start time of the oil spill and its degree of uncertainty is of substantial significance to the simulation and prediction of the transport of marine oil spills and has a significant impact on the planning of the emergency response to the spilled oil.

### 3.5. Influence of Uncertainty in the Spill Site

**Figure 14:**
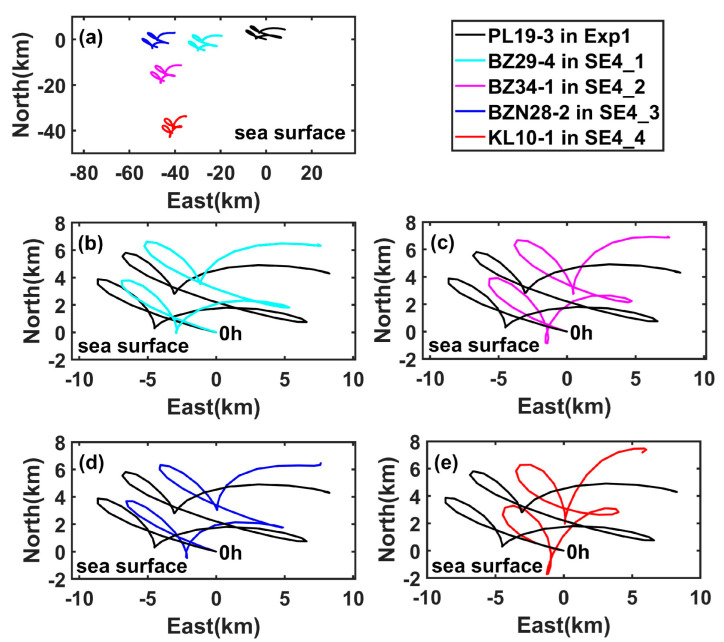
(**a**) Simulated centroid trajectory at the sea surface (relative to the position of PL19-3) in Exp1 (black line), SE4_1 (cyan line), SE4_2 (magenta line), SE4_3 (blue line), and SE4_4 (red line); (**b**) Comparison between the simulated centroid trajectory (relative to its own platform position) at the sea surface in Exp1 (black line) and that in SE4_1 (cyan line); (**c**–**e**) are similar to (**b**), but for SE4_2, SE4_3, and SE4_4.

When the underwater oil spills occur on different oil platforms, the centroid trajectories of the oil spill at the sea surface have similar trend (Figure 14a), mainly because these oil platforms are close to each other. From Figure 14b to Figure 14e, the simulated centroid trajectories, which are relative to the corresponding platform in the sensitivity experiments numbered with prefix ‘SE4′, are different from those in Exp1; besides, the east-west ranges of the drift trajectories in SE4_1, SE4_2, and SE4_3 are approximately the same, but the east-west range in SE4_4 is much narrower and the south-north range is much larger. The distances between the final centroid point at 48 h and the corresponding oil platform in Exp1, SE4_1, SE4_2, SE4_3, and SE4_4 are 9.00 km, 9.81 km, 10.12 km, 10.00 km, and 9.19 km, respectively, indicating that the distance of oil spill drift from the oil platform makes little difference, as the marine and atmospheric environments are relatively similar in this area. In addition, the centroid trajectories of the oil spilled from different oil platforms do not intersect with each other within 48 h. Therefore, the uncertainty of the spill site has little impact on the pattern of the simulated oil spill trajectory at the sea surface, and has large influence of the final position of the oil spill centroid point; in addition, it is possible to identify the oil spill platform from the actual observations of the oil film within 48 h.

## 4. Discussion

Oil pollution has an adverse influence on the marine environment, ecology, and regional sustainable development capacity [35]. The numerical model of the underwater oil spill is the most effective method to predict the transport and fate of the spilled oil, but the accuracy of the predicted results using the numerical model is significantly affected by the model uncertainty [15,17,18,19,22]. As indicated by Gonçalves et al. [5], the uncertainties in the oil spill location, the amount of the spilled oil, the flow rate of the oil, the oil droplet size distribution, the ocean current, the subgrid-scale parametrization, the wind drag at the surface, the oil degradation, and other parameters would influence the simulated and predicted fate of the spilled oil. It is necessary to investigate the influence of model uncertainties [6,14,16,20,21]. The start time and spill site are the basic temporal and spatial information for simulating the underwater oil spill, and the wind and current are the necessary input for the underwater oil spill model. Therefore, the influence of the uncertainties in these parameters on the transport of underwater spilled oil from the PL19-3 platform are investigated in this study.

Elliott and Jones [17] indicated that the accurate wind forecast results were the single most important factor for predicting the transport of an oil slick, but for the underwater oil spill the current is much more important than the wind, as shown in this study. Unlike the oil spill at the sea surface, the underwater spilled oil not only reaches the sea surface but also stays underwater. For the underwater oil spill, the numerical simulation that begins from the observed oil slick at the sea surface at some time would ignore the influence of the underwater oil, and the simulated results may significantly deviate from the actual situation. So, it is necessary to simulate and predict the transport and fate of the underwater spilled oil for the whole accident. However, the start time and spill site of the underwater oil spill are hard to obtain, and are usually estimated based on experience, resulting in a lot of uncertainty, especially in the actual emergency forecasting. The uncertainties in the start time and spill site are discussed in this study, which make it an important supplement to and improvement on Li et al. [15], who only studied the impact of error in ocean current on the transport of underwater spilled oil.

With the progress of observation technology and data processing methods, there are many more means to obtain information on the spilled oil. For example, Dong et al. [35] developed the first global map of oil slicks by analyzing the Sentinel-1 images from 2014–2019, which provided the potential observations for calibrating the numerical model of oil spills and improving the accuracy of prediction. Based on the precious observation data of oil slicks at the sea surface or the underwater spilled oil, the start time and spill site of the underwater oil spill may be inversely identified, and the uncertainties of the model parameters reduced; this would be important to further improve the accuracy of numerically predicting the transport and fate of oil spill, and to provide information for the risk assessment and emergency response during the oil spill accidents, which will be investigated in our future work.

## 5. Conclusions

Based on a three-dimensional underwater oil spill model validated by satellite images of the oil slick at the sea surface, the Penglai 19-3 oil spill accident in the Bohai Sea was simulated; in addition, several sensitivity experiments were set up to investigate the influence of model uncertainties on the simulated oil spill trajectory at the sea surface, the centroid position and sweeping area at the sea surface and underwater, and the sweeping volume. When the maximum random error in wind is 30%, the error expectation of centroid shift at the sea surface is 44.08 times of that in the benchmark experiment, while the uncertainty in the wind has little effect on the simulated underwater results, as the wind mainly drives the oil drift at the sea surface. When the uncertainty in the current is considered together with the uncertainty in the wind, the influence on the simulated transport of the underwater spilled oil is significantly greater than with only the uncertainty in the wind, showing the importance of the current for the accurate simulation of an underwater oil spill. As the tidal current is the significant constituent of the current in the Bohai Sea and its periodicity, the start time of the oil spill has a significant influence on the transport of the oil; in detail, an uncertainty of 24 h can cause 1.19 (1) times larger than before displacement of the oil spill centroid point (sweeping area) at the sea surface. The uncertainty of the spill site has little impact on the pattern of the simulated oil spill trajectory at the sea surface, and has large influence on the final position of the oil spill centroid point.

When considering the numerical simulation and prediction of actual oil spill accidents, the model uncertainty should be considered adequately, which is of substantial significance in improving the prediction ability of underwater oil spills. This study concludes that the current and the start time of an oil spill have significant influence on the transport of underwater spilled oil, while wind would mainly impact the surface results; in addition, it is possible to identify the oil spill platform from the actual observations of the oil slick at the sea surface obtained shortly after the oil spill has occurred.

## Figures and Tables

**Table 1:** Detailed model settings of the numerical experiments.

No	Maximum Random Error in Wind	Maximum Random Error in Current	Starting Time (Relative to That in Exp1)	Spill Site
Exp1	0	0	0 h	PL19-3
SE1_1	10%	0	0 h	PL19-3
SE1_2	20%	0	0 h	PL19-3
SE1_3	30%	0	0 h	PL19-3
SE2_1	10%	10%	0 h	PL19-3
SE2_2	20%	20%	0 h	PL19-3
SE2_3	30%	30%	0 h	PL19-3
SE3	0	0	1 h to 24 h	PL19-3
SE4_1	0	0	0 h	BZ29-4
SE4_2	0	0	0 h	BZ34-1
SE4_3	0	0	0 h	BZN28-2
SE4_4	0	0	0 h	KL10-1

## Data Availability

Not applicable.
